# Level of unintended pregnancy among reproductive age women in Bahir Dar city administration, Northwest Ethiopia

**DOI:** 10.1186/s13104-018-4016-z

**Published:** 2018-12-14

**Authors:** Eleni Admasu, Alemtsehay Mekonnen, Tesfaye Setegn, Gedefaw Abeje

**Affiliations:** Department of Reproductive Health, School of Public Health, College of Medicine and Health Sciences, Bahir Dar University, Bahir Dar, Ethiopia

**Keywords:** Unintended pregnancy, Bahir Dar, Mass media

## Abstract

**Objective:**

The objective of this study was to determine the prevalence and determinants of unintended pregnancy among reproductive age women in Bahir Dar town, Northwest Ethiopia.

**Result:**

The prevalence of unintended pregnancy was 15.8% (95% CI 13.8%–17.7%). Single women (AOR 0.18; 95% CI 0.08–0.40), women living away from their husband (AOR 4.18; 95% CI 2.64–6.61) and women with no access/exposure to mass-media (AOR 1.89; 95% CI 1.13–3.15) were more likely to have unintended pregnancy compared to their counter parts.

**Electronic supplementary material:**

The online version of this article (10.1186/s13104-018-4016-z) contains supplementary material, which is available to authorized users.

## Introduction

Unintended pregnancy is identified as pregnancy either mistimed or unwanted at a time of conception regardless of women’s contraceptive use [1]. A pregnancy is assumed mistimed if a woman became pregnant at a time when she did not want to. On the other hand, pregnancy is assumed unwanted if the woman did not intend to ever become pregnant, or if the pregnancy occurred when she wanted to have no more children [2]. Some of the leading causes of unintended pregnancies include not using contraceptives, contraceptive failure and less commonly rape [3].

Unintended pregnancy is a public health problem which affects maternal and child health [4, 5]. In 2012, about 40% of the estimated 213 million pregnancies were unplanned [6]. In Sub-Saharan Africa, approximately 14 million unintended pregnancies occur every year [7]. According to the 2011 Ethiopian Demographic and Health Survey (EDHS) report, 32% of pregnancies at the time of survey were reported unintended [8]. Other studies in the Eastern and Southern part of Ethiopia also showed high proportion of unintended pregnancy [9–11].

Although unintended pregnancy has social, cultural and health impacts, especially for the mother and the fetus, little is known about the prevalence and associated factors of unintended pregnancy among women in Bahir Dar. Therefore, the aim of this study was to determine the prevalence and identify determinants of unintended pregnancy among women in Bahir Dar town.

## Main text

### Methods

A community based cross-sectional study was conducted among reproductive age women in Bahir Dar city administration. The city administration is located 565 km Northwest of Addis Ababa, the capital city of Ethiopia. Bahir Dar is located at 565 km from Addis Ababa, capital of Ethiopia and administratively divided into 9 urban and 12 rural Kebeles (the smallest administrative unit in Ethiopia). There are 10 health centers and three hospitals (one government and two private hospitals), which provides maternal and child health services including family planning service [12].

The study included 680 pregnant women or women with under-1 year old child at the time of the study. Sample size was determined using single population proportion formula considering 95% confidence level, 5% margin of error and 27.9% estimated prevalence of unintended pregnancy (9). Finally after adding 10% of the calculated sample size for non-responses and using design effect of 2, the sample size was determined to be 680. From the total of 10 kebeles, 7 Kebeles were selected randomly. The total sample size was proportionally allocated to each kebeles and individual women were selected using systematic random sampling technique using the maternal registration book (prepared by community health extension workers) as a sampling frame.

The data were collected by five trained nurses (supervised by two midwives) using a structured interviewer administered questionnaire (Amharic version-local language). The questionnaire was developed after reviewing relevant literatures (Additional file 1). Pretest was done to check the validity and consistency of the questionnaire.

Data was entered, cleaned and analyzed using SPSS version 20 statistical software. Both descriptive and analytical analyses were carried out. The woman was considered having unintended pregnancy if she reported if her recent birth or current pregnancy was mistimed or unwanted. Exposure to mass-media was considered if the woman had TV or radio at home. Bivariate and multivariable logistic regression analyses were carried out to identify the presence of statistical significance between the outcome variable with each independent variable. Statistical differences were considered at p < 0.05 and the strength of association was assessed by odds ratio (OR) with the respective confidence intervals.

Ethical clearance was obtained from the Ethical Review Committee (ERC) of Bahir Dar University, College of Medicine and Health Sciences. Permission letter was obtained from Amhara National Regional State (ANRS) Health Bureau and Bahir Dar city administration health office. Verbal consent was obtained from each study participants after they were informed about the purpose and objective of the study. They were also assured that they have the right to decline from answering the whole or parts of question they assume private.

### Result

#### Socio demographic characteristics of women

From a total of 680 women, 674 (91% response rate) were interviewed and included in the analysis. Six women were not found at the time of the study even with repeated attempts. Fifty-five questionnaires were not included in the analysis because there were inconsistencies.

The mean (± SD) age of the women was 28.2 (± 5.8) years. Majority (84%) of women were urban residents. About fifty-six percent (56.4%) of the respondents were married of which 57.7% reported that their marriage was arranged by their family. More than eighty-three percent (83.5%) of women were Orthodox Christians and 97.6% were from Amhara ethnic group. About 60% of women reported that they were housewives. Nearly 40% women did not attend formal education (Table 1). In our study, 88.7% and 94.3% of the women reported that they were autonomous to visit their family and health institutions respectively. Ninety-eight percent (98%) women reported that they have access to health institution with family planning service within 1 h walking distance.

**Table 1:** Socio demographic characteristics of women in Bahir Dar city administration, February–March, 2015

Variable	Number (%)
Age in years	
≤ 25	243 (39.3)
26–34	258 (41.7)
≥ 35	118 (19.1)
Marital status	
Single	66 (10.7)
Married	535 (86.4)
Divorced/widowed	18 (2.9)
Religion	
Orthodox	517 (83.5)
Catholic and protestant	21 (3.4)
Muslim	81 (13.1)
Mother’s occupation	
Housewife	375 (60.6)
Employee	92 (14.9)
Small business	75 (12.1)
Other*	77 (12.4)
Mother’s educational status	
No formal education	243 (39.3)
Primary	234 (37.8)
Secondary and above	142 (22.9)
Husband occupation	
Farmer	99 (16.0)
Employee	208 (33.6)
Merchant	189 (30.5)
Other*	123 (19.9)
Husband education	
No formal education	194 (31.3)
Primary	233 (37.6)
Secondary and above	192 (31.0)

* Other = student, daily laborer

#### Obstetric characteristics of respondents and level of unintended pregnancy

In our study, majority (89.3%) of the participants got pregnant for the first time at after 18 years old. Sixty-nine percent (69.1%) of women were multipara. About 86% of women visited health facility for the current pregnancy while 10% of women reported that they had history of abortion. The study also showed that (48.6%) of women (out of the total women who had under-1 year child) gave birth at health institutions. Seventy-five percent (75%) of women reported ever use of contraceptives. But only 17.1% reported ever use of long acting contraceptive methods. In this study, the prevalence of unintended pregnancy was found to be 15.8% (95% CI 13.8%–17.7%).

#### Factors associated with unintended pregnancy

Bivariate and multivariable logistic regression analysis was done with 95% CI to identify determinants of unintended pregnancy in Bahir Dar city administration. Nineteen variables were entered in the bivariate and multivariable logistic regression analysis but only marital status, living arrangement at the time of interview and availability of mass media at home remained significant in the bivariate and multivariable analysis (Table 2).

**Table 2:** Determinants of unintended pregnancy in Bahir Dar town, Northwest Ethiopia, February–March, 2015

Variable	Current/recent pregnancy		COR (95% CI)	AOR (95% CI)
	Intended	Unintended		
Age in years				
≤ 25	196	47	1.33 (0.74–2.41)	1.29 (0.51–3.26)
26–34	225	33	0.85 (0.44–1.52)	0.88 (0.39–1.95)
≥ 35	100	18	1.0	1.00
Current living condition				
With husband	433	53	1.00	1.00
Living away from husband	88	45	4.18 (2.64–6.61)	3.05 (1.46–6.34)
Family size				
≤ 6	496	92	0.77 (0.31–1.94)	0.32 (0.10–1.03)
> 6	25	6	1.00	1.00
Residence				
Urban	431	86	1.50 (0.79–2.85)	2.16 (0.76–6.18)
Rural	90	12	1.00	1.00
Mother’s occupation				
Housewife	326	49	1.00	1.00
Employee	82	10	0.81 (0.39–1.67)	0.61 (0.23–1.66)
Small business	64	11	1.14 (0.56–2.32)	1.80 (0.73–4.43)
Other	49	28	3.80 (2.19–6.61)	2.06 (0.94–4.53)
Mother’s educational status				
No formal education	197	46	1.72 (0.94–3.13)	1.63 (0.65–4.08)
Primary	199	35	1.29 (0.70–2.41)	0.61 (0.25–1.50)
Secondary and above	125	17	1.00	1.00
Autonomy to visit family				
Autonomous	462	87	1.00	1.00
Not autonomous	59	11	0.99 (0.50–1.96)	0.39 (0.14–1.11)
Distance of HF from home				
≤1 h	510	97	1.00	1.00
>1 h	11	1	0.48 (0.06–3.75)	0.99 (0.10–9.42)
Mass media available at home				
Yes	441	73	1.00	1.00
No	80	25	1.89 (1.13–3.15)	2.81 (1.27–6.23)
Marital status				
Single	31	35	1.00	1.00
Married	480	55	0.10 (0.06–0.18)	0.18 (0.08–0.40)
Widowed/divorced	10	8	0.71 (0.25–2.02)	0.55 (0.60–3.10)
Gravidity				
Primigravida	176	48	1.00	1.00
Multigravida	345	50	0.53 (0.34–0.82)	0.68 (0.34–1.38)

In this study married women were 82% less likely (AOR 0.18; 95% CI 0.08–0.40) to have unintended pregnancy compared single women. Women who were living away from their husband were about 4 times (AOR 4.18; 95% CI 2.64–6.61) more likely to have unintended pregnancy compared to women who reported that they were living with their husband. Availability of mass media at home was an important predictor of unintended pregnancy. Women who reported that there was no mass media at home were about two times (AOR 1.89; 95% CI 1.13–3.15) more likely to have unintended pregnancy compared to women who reported presence of mass media at home.

### Discussion

In our study, the prevalence of unintended pregnancy was found to be 15.3 (95% CI 13.8–17.7%) for the current pregnancy or for the last birth women had. This finding is lower that national figure reported in the Ethiopian Health and Demographic Data [8]. Similarly, our finding is lower when compared with other research findings conducted in Ethiopia [11, 13]. This difference could be due to the difference in the scope, sample size, design and study period of the studies. For example, the national survey (EDHS, 2011) included both women from urban and rural settings proportionally. However, in our study, majority of participants were urban residents who could have better access to family planning services and including information.

Although the level of unintended pregnancy seems low in this study, compared to other findings in Ethiopia, different determinant factors could circumscribe the even and impact maternal health and neonatal/child health much. In this study, marital status of woman (being married) (AOR 0.18; 95% CI 0.08–0.40), was found to be a significant factor for unintended pregnancy where married women were 82% less likely to have unintended pregnancy compared to single women. In the same fashion, studies conducted in Gelemso and Debremakos [14, 15] showed that married women have lesser risk to experience unintended pregnancy. This evidences showed the reverse that single women have higher risk of having unintended pregnancy. The probable reason for this could be that single women are more likely to have unplanned sexual activity and ashamed of using contraception, which leads them to have unintended pregnancy.

In the study, women who reported living away from their husband were 4 times (AOR 4.18; 95% CI 2.64–6.61) more likely to have unintended pregnancy compared to women who reported living with their husband at the time of survey. The reason for this is that women who live away from their husband do not use contraceptives regularly due to irregular sexual intercourse. Similarly, women who reported no access to mass media at home were more likely (AOR 1.89; 95% CI 1.13–3.15) to have unintended pregnancy compared to women who reported access. The reason for this may be women with access to mass media have better information about contraceptives and more likely to use family planning services. In conclusion, significant proportion of women have unintended pregnancy in Bahir Dar town although the magnitude is lower than previous studies done in different parts of Ethiopia. Single women, women who live away from their husband and women with no access to mass media are more likely to have unintended pregnancy.

## Limitations of the study

Although the study identified some of the determinants of unintended pregnancy, the result may be affected by social desirability bias. Women tend to report that their pregnancy or recent birth is intended although the reality is different. The other limitation of the study is that this study may have missed women with unintended pregnancy who already aborted their pregnancy. This may underestimate the level of unintended pregnancy.

## Additional file

**Additional file 1.** Questionnaire prepared to study determinants of unintended pregnancy among women in Bahir Dar city.
