# Supplementary material for: Level of unintended pregnancy among reproductive age women in Bahir Dar city administration, Northwest Ethiopia
Source: BMC Res Notes. 2018 Dec 14;11:891. doi: 10.1186/s13104-018-4016-z (PMC6295029; doi:10.1186/s13104-018-4016-z)
Supplement: Supplementary file 1 — Additional file 1. Questionnaire prepared to study determinants of unintended pregnancy among women in Bahir Dar city. [file 13104_2018_4016_MOESM1_ESM.docx]

Bahir Dar University College Medicine and Health Science

School of Public Health

Questionnaire prepared to study prevalence of unintended pregnancy and associated factors among pregnant women and women Who Have less than one year children, Bahir Dar town North West Ethiopia, 2014.

Instruction: write or circle what the woman said.

**Part one**: - socio-demographic characteristics

| code | Question | Answer/Options |
| --- | --- | --- |
| 101 | Age | _______________years |
| 102 | Marital status | 01.single  02.single live with her families  03.married  04.widowed  05. Divorced  97.others |
| 103 | If married marital condition | 01.family  02.love  97.others |
| 104 | Marital age | ___________________years |
| 105 | Age difference with husband | ___________________years |
| 106 | Age at first pregnancy | ___________________years |
| 107 | Currently, live with whom? | 01.with my husband  02.with my husband’s family  03.with my family  97.others |
| 108 | Family size | __________________ |
| 109 | Residence | 01.urban  02.rular |
| 110 | Religion | 01.Orthodox  02.Catholic  03.Protestant  04.Muslim  97.others |
| 111 | Ethnicity | 01.Amhara  02.Tigrie  03.oromo  04, Agew  97.others |
| 112. | Occupation | 01.houswife  02.GOV’T employee  03.NGO employee  04.Merchant  05.dailylabor  97.othes |
| 113 | Her educational status | 01.illitrate  02.able to read and write  03.______________grade  04.colledge and above |
| 114 | Monthly income | __________________birr |
| 115 | Husband age | ____________________years |
| 116 | Husband occupation | 01.farmer  02.GOV’T employee  03.NGO employee  04.selfeployee  05.dailylabor  97.others |
| 117 | Husband’s educational status | 01.illitrate  02.able to read and write  03.________________grade  04.colledge and above |
| 118 | Distance from health facilities | 1.Near  2.Medium  3. Far |
| 119 | Media | 01.radio  02.ETV  03.none  97.others |

**Part 2**:- Obstetrical and gynecological characteristics

| Code | Question | Answer/options |
| --- | --- | --- |
| 201 | Gravidity | __________________ |
| 202 | Parity | __________________ |
| 203 | Currently no. of alive birth | __________________ |
| 204 | Stillbirth | __________________ |
| 205 | Abortion | __________________ |
| 206 | Did you have ANC follow up for the previous pregnancy? | 01. yes,  02.no |
| 207 | If your answer is ‘yes’ for Q. No ‘206 ‘ How many times | 01.--------------times |
| 208 | Place of delivery for previous pregnancy | 01.home  02.health center  03.hospital  97.others |
| 209 | Current pregnancy condition | 01.wanted and planed  02.mistimed  03.unwanted |
| 210. | If the pregnancy is/was ‘unwanted’, why? | 01.not supported by my husband  02.I am a student  03.I am kid  04.fanancial problem  05.contraceptive failure  97.othres |
| 211 | If your answer is ‘mistimed’ why? | ________________ |
| 212 | Did You know Contraceptive? | 01.yes  02.no |
| 213 | Had you ever used any contraceptive? | 01.yes  02.no |
| 214 | If you used what type of contraceptive? | 01.pills  02.DEPO  03.implant  97.others |
| 215 | Had you used correctly? | 01.YES  02.NO |
| 216 | If you didn’t use any type of contraceptive why? | ____________________________ |
